# Advances in engineering nanoparticles for magnetic particle imaging (MPI)

**DOI:** 10.1126/sciadv.ado7356

**Published:** 2025-01-08

**Authors:** Ambar C. Velazquez-Albino, Eric Daniel Imhoff, Carlos M. Rinaldi-Ramos

**Affiliations:** ^1^ Department of Chemical Engineering, University of Florida, Gainesville, FL 32611, USA.; ^2^ J. Crayton Pruitt Family Department of Biomedical Engineering, University of Florida, Gainesville, FL 32611-6131, USA.

## Abstract

Magnetic particle imaging (MPI) is an emerging imaging modality with exciting biomedical applications, such as cell tracking, blood pool imaging, and image-guided magnetic hyperthermia. MPI is unique in that signal is generated entirely by synthetic nanoparticle tracers, motivating precise engineering of magnetic nanoparticle properties including size, shape, composition, and coating to address the needs of specific applications. However, success in many applications and in clinical transition requires development of high-sensitivity and high-resolution tracers, for which there is considerable room for improvement. This review summarizes recent advancements in MPI tracer synthesis and compares reported tracers in terms of sensitivity and resolution. In making these comparisons, we point out inconsistencies in reporting of MPI tracer properties. To overcome this challenge, we propose a list of properties to standardize characterization and reporting of new MPI tracers and improve communication within the field.

## Teaser

Controlling nanoparticle magnetism and thorough characterization are key to advancing magnetic particle imaging.

## INTRODUCTION

Modern medical science has progressed rapidly for the benefit of humanity; however, proper diagnosis remains a necessity before treatment (*1*). Medical imaging has a rich history of using contrast agents and tracers to facilitate diagnosis and treatment; however, efficacy is heavily dependent on the quality of the image. This has led to major advancements in medical imaging over the past few decades, including the development of new techniques that enhance the scope of one method without necessarily replacing others. For example, computed tomography (CT) is often integrated with positron emission tomography to provide detailed information in the detection and monitoring of cancer, heart disease, and brain disorders (*2*). While these tools have proven invaluable, challenges remain in overcoming limitations in sensitivity, resolution, and dose limiting toxicity. For example, increased radiation exposure has been associated with increased cancer risks in adults and particularly in children. In 2007, it was estimated that about 1 to 2% of cancer in the US was attributable to radiation from CT scans, a technique which can entail large doses of radiation (*3*). This underscores the need for new and complementary imaging tools.

Magnetic nanoparticles are of great interest because of their biocompatibility, their tunable size and surface chemistry, and their magnetic response, which results in many unique applications (*4*, *5*). They have shown tremendous potential as magnetic resonance imaging (MRI) contrast agents (*6*), thermal cancer therapy agents (*7*), and triggered drug release vehicles (*8*) and in rewarming of cryopreserved organs (*9*, *10*). Magnetic particle imaging (MPI) was first introduced 2005 as a novel noninvasive and tomographic medical imaging modality, wherein signal arises solely from the nonlinear dynamic magnetization response of superparamagnetic nanoparticles, enabling longitudinal, radiation-free, unambiguous, and sensitive quantification of their biodistribution (*11*). These features make MPI a promising tool in the medical imaging toolbox, especially for applications that require quantitative longitudinal tracking or repeated imaging. For example, cell therapies have rapidly evolved for personalized medicine in cancer; however, most are at the research and translational phases because of major challenges such as safety and cost-benefit considerations (*12*). MPI can enhance current cell therapy development by enabling repeated quantitative image-based tracking to obtain information on their location, trafficking, and persistence at target sites, which may correlate with patient clinical response and could be used to predict patient outcome (*13*). Similarly, MPI can provide valuable information to aid in development of other biomedical applications of magnetic nanoparticles.

This review is divided into five main parts. The first part covers the basic theory of MPI, including predictions derived from the Langevin model to describe MPI performance and deviations from this simple model. The second part provides an overview of methods of magnetic nanoparticle synthesis, postsynthesis modifications, and characterization methods with an emphasis on work related to MPI, rather than the much broader literature on magnetic nanoparticle synthesis and characterization. The third part provides an overview of commercially available tracers, typically developed for other applications, that have found use in MPI. The fourth part provides a critical review of recent efforts to develop tracers for MPI consisting of iron oxide nanoparticles, substituted ferrites, and other compositions. In the final part, the review provides a comparison of tracer performance for tracers for which sufficient data were reported to support such comparisons, limitations of past work in terms of inconsistent or incomplete characterization are highlighted, and recommendations are made to improve rigor in characterizing and reporting the properties of new MPI tracers.

## MAGNETIC PARTICLE IMAGING

The signal generated in MPI relies on the nonlinear dynamic magnetization of superparamagnetic iron oxide nanoparticle (SPION) tracers in a time-varying magnetic field and is proportional to the tracer mass, providing quantitative visualization of their distribution, without ionizing radiation. Because the signal is solely generated by the tracer, there is no tissue attenuation or background, enabling deep tissue imaging, which can be advantageous in certain areas of the body that might be limited in other imaging modalities. These characteristics render MPI an ideal method for noninvasive quantification of SPIONs in biological environments, as it can form three-dimensional (3D) images of tracer distribution. This highly sensitive imaging modality has numerous potential clinical applications, such as in cell tracking (*14*–*24*), blood pool imaging (*18*–*21*, *23*), and traumatic brain injury (*22*). Furthermore, MPI has the capability of synergistically enhancing development of already existing applications of magnetic nanoparticles such as magnetic hyperthermia (*7*, *25*), drug delivery (*26*), magnetically triggered drug release (*27*–*29*), magnetic targeting (*8*, *30*–*32*), and rewarming of cryopreserved organs (*9*).

Even though MPI is a sensitive and quantitative imaging modality, we have yet to fully exploit its potential because of a lack of nanoparticle tracers tailored for high sensitivity and resolution. To fully maximize MPI capability and advance the field, optimization of tracers is crucial. MPI relies on the dynamic magnetization of tracers to generate a signal; therefore, their magnetic properties and interactions are key to tune and enhance MPI performance for a growing number of applications. In addition, more sensitive tracers with improved resolution would allow for the use of lower magnetic field gradients during signal acquisition, reducing capital and operational costs to aid in development of clinical equipment.

### Basic theory of MPI

**Fig. 1.**
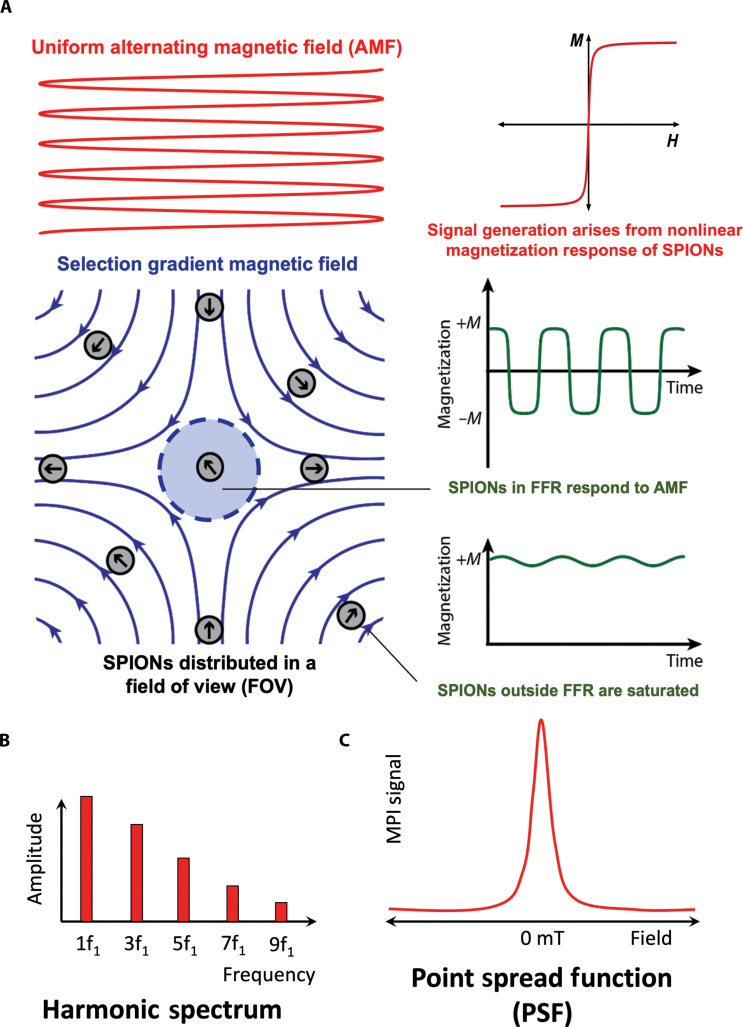
Fundamental concepts of signal generation in MPI. (**A**) Magnetic fields in the FOV and SPION response in the FFR. (**B**) Harmonic spectrum from system matrix image reconstruction. (**C**) PSF from x-space image reconstruction. Figure adapted with permission from (*5*).

Currently, MPI images are generated by small-bore preclinical MPI scanners that are accessible for animal research, while efforts are underway to develop larger clinical scanners suitable for human use. MPI signal is generated from the nonlinear response of SPIONs in the field of view (FOV) of the scanner subjected to a uniform alternating magnetic field (AMF) (Fig. 1A). The key principle to obtain MPI images is the superposition of a nonuniform selection gradient magnetic field, which forms a field-free region (FFR), where SPIONs can respond to the AMF, and which saturates the nanoparticles outside of that region (Fig. 1A). SPIONs in the FFR exhibit a magnetization response that results in an induction of a voltage in a receiving coil, while the magnetic response of particles outside the FFR is negligible as they are saturated (Fig. 1A). In MPI scanners, the FFR is moved across the FOV to obtain the signal of the magnetic tracers inside the scan volume as a function of 3D space, which is then processed using either system matrix reconstruction or x-space reconstruction to generate an image (*5*).

The first image reconstruction method used was system matrix reconstruction (*11*, *33*). This method uses a system matrix to precharacterize the signal response of SPIONs, which contains the Fourier harmonics (Fig. 1B) of the tracer response under typical MPI excitation fields at multiple possible locations of a point source. Reconstruction involves matrix inversion techniques, such as singular value decomposition, which become complex because of the dense nature of the system matrix (*11*, *34*). The major challenges of system matrix reconstruction are the time needed to reconstruct the images and the precharacterization scans required for calibration, which are tracer and environment specific (*34*–*36*).

The second image reconstruction method developed was x-space reconstruction, introduced by Goodwill and Conolly in 2010 (*37*), and for which tracer performance is typically associated to a so-called point spread function (PSF; Fig. 1C). X-space reconstruction increases the speed and robustness of MPI image reconstruction through a two-step process of velocity compensation followed by gridding into the direct position of the FFR. A major advantage of the x-space approach is the possibility of real-time image reconstruction, as well as not requiring precharacterization, matrix inversion, or modeling of the in vivo environment (*35*, *37*). For an in-depth comparison of these reconstruction methods, we refer the reader to reviews focusing on MPI reconstruction (*38*, *39*).

Regardless of the image reconstruction method used, the tracer signal arises because of the nonlinear dynamic magnetization of the particles. While dynamic magnetization of magnetic nanoparticles can be complex, a simple model assuming fast nanoparticle superparamagnetic response can provide insight into the relationship between nanoparticle properties and MPI performance (sensitivity and resolution). The Langevin model describes the equilibrium magnetization (*M*) of superparamagnetic nanoparticles as a function of applied magnetic field (*H*) (Fig. 1A). This model assumes the system consists of a number density (*N*) of noninteracting single-domain superparamagnetic nanoparticles with a magnetic moment that instantaneously aligns with the applied magnetic field (i.e., with negligible relaxation time) (*37*, *40*). In the x-space approach to MPI reconstruction, the PSF (Fig. 1C) is related to the time derivative of the *M*(*H*) curve and can be approximated using models like the Langevin function (*37*). The sensitivity is proportional to the intensity (*I*), the height of the PSF peak, and the full width at half maximum (FWHM) corresponds to resolution. The following equations can be derived from the Langevin model, where μ_0_ is the vacuum permeability, $k_{B}$ is the Boltzmann constant, *T* is temperature, $M_{\text{sat}}$ is the saturation magnetization, *D* is the particles’ diameter, and *G* is the magnetic field gradient (*37*, *41*). However, these equations are at best proportional to the performance

$$I=\frac{{N\pi M}_{\text{sat}}D^{3}}{18}$$ (1)

$$\text{FWHM}=\frac{24k_{B}T}{\mu_{0}{\pi M}_{\text{sat}}{\mathrm{GD}}^{3}}$$ (2)

On the basis of this simple model, optimizing MPI performance consists of increasing sensitivity (proportional to intensity, *I*) and improving resolution by decreasing the FWHM, which theoretically should be achieved by increasing nanoparticle diameter (*D*) and saturation magnetization ($M_{\text{sat}}$). The saturation magnetization is an intrinsic parameter of the material used, which should be measured and reported when optimizing tracers, as different phases, grains, and defects in a material can affect the saturation magnetization. In the literature, measurements of saturation magnetization lower than the desired bulk material are often alluded to the existence of a “magnetically dead layer.” This further introduces the concept of a so-called magnetic diameter, which is representative of the strength of the magnetic dipole of the particles. Several publications suggest that the magnetic diameter often differs from the physical diameter [i.e., that obtained by transmission electron microscopy (TEM)], either because of the effect of a magnetically dead layer or because of other causes, like formation of multiple magnetic domains within a single particle (*41*–*44*). While these tracer properties are key, Eq. 2 also suggests that resolution can be improved by increasing the magnetic field gradient strength. However, this substantially increases the cost of the scanner and introduces safety concerns. Therefore, clinical scale-up considerations limit the range of magnetic field conditions that can be used in MPI.

### Deviations from the Langevin model

The Langevin theory of MPI covered above assumes that SPIONs instantly align with the applied magnetic field. Deviations from this assumption degrade MPI performance because of the delay in magnetization (*45*). Magnetic relaxation describes how SPIONs respond to a change in applied magnetic field, through processes governed by the so-called Néel and Brownian mechanisms. The Néel mechanism refers to magnetic dipoles rotating within the particle to align with the field, while the Brownian mechanism consists of physical particle rotation to align their dipole with the local magnetic field. In principle, nanoparticles undergo relaxation by the mechanism with the shortest characteristic time. This relaxation time is in turn highly dependent on particle size, composition, crystal phase, thickness of coating, and temperature. The Néel, τ_N_, and Brownian, τ_B_, relaxation times are (*46*)

$$\tau_{N}=\tau_{0}\text{exp}\left(\frac{{\mathrm{KV}}_{C}}{k_{B}T}\right)$$ (3)

$$\tau_{B}=\frac{3\eta V_{h}}{k_{B}T}$$ (4)

In Eqs. 3 and 4, τ_0_ is a time constant on the order of 10^−10^ s, η is the viscosity of the fluid, *K* is the magnetic anisotropy constant, *V*_C_ is the magnetic core volume, and *V*_h_ is the hydrodynamic volume of the particles. It is important to point out that Eqs. 3 and 4 are strictly valid in the limit of negligible applied magnetic field strength, which is not the case under MPI excitation field conditions. Also, both Brownian and Néel relaxation times depend heavily on the magnitude of the external applied field (*47*). Still, we use these equations to illustrate the complexity of SPION magnetic relaxation response. Even though both time constants have a volume dependence, it is much stronger for the Néel relaxation time constant, as it is an exponential dependence on the cube of the diameter. In general, the relaxation time increases as particle size increases, deviating from the Langevin model assumption of negligible relaxation time. When the relaxation time becomes comparable to the timescale of the MPI excitation field, there is a loss in signal intensity and resolution (*45*).

**Fig. 2.**
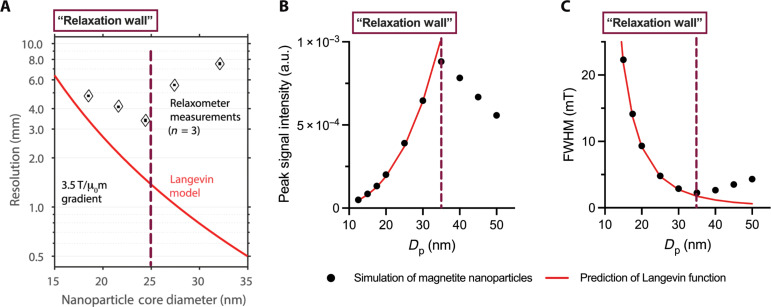
Comparison of MPI performance and predictions of the Langevin function elucidate the existence of a relaxation wall, after which relaxation effects degrade MPI performance. As a function of the physical core diameter, (**A**) resolution (millimeters) from relaxometry measurements compared to the predicted resolution from the Langevin model shows a rapid increase in deviation at a core diameter of 25 nm adapted with permission from (*48*). Copyright Institute of Physics and Engineering in Medicine. Reproduced by permission of IOP Publishing Ltd. All rights reserved. Langevin function predictions are also compared as a function of physical diameter to (**B**) peak signal intensity, and (**C**) resolution as FWHM in milliTesla obtained from computational modeling of x-space MPI physics redrawn using data from (*49*) Copyright IOP Publishing. Reproduced with permission. All rights reserved. The relaxation wall, the physical diameter at which relaxation effects deviate performance from the predictions of the Langevin function, is highlighted as a vertical red dashed line.

The barrier of improving MPI performance due to increasing relaxation effects has been named in the literature as the “relaxation wall.” A study comparing resolution from relaxometry measurements of tracers of core sizes between 18 and 32 nm showed that increasing relaxation eventually opposes the expected improvement from the Langevin model, leading to worsening of resolution for core sizes above 25 nm (Fig. 2A) (*48*). Computational modeling of x-space MPI physics considering relaxation effects by Zhao *et al.* (*49*) confirms the inevitability of a relaxation wall, showing good agreement with the Langevin model for particles under a critical diameter and worsening performance above that critical diameter due to increasing relaxation (Fig. 2, B to D). This behavior is consistent, as the trend is observed when modeling the signal intensity (Fig. 2B) and resolution (Fig. 2C). We have identified the relaxation wall in Fig. 2 with a vertical red dashed line to guide the reader, and we note that it corresponds to different physical diameters for the experimental and computational studies shown. The exact diameter at which relaxation effects start to degrade performance cannot be generalized, as it is dependent on the tracer properties, such as the magnetic core volume, the shape and crystal defects that can affect the effective anisotropy, the hydrodynamic diameter, and even their environment, such as surface coating, fluid viscosity, and temperature. However, the conclusion is clear: Increasing relaxation effects due to increasing tracer size halt improvement of MPI performance (*45*, *48*–*51*).

The Néel relaxation time in Eq. 3 also has an exponential dependence on magnetic anisotropy (*K*), which is influenced by factors such as shape, size, and crystal structure. The effect of anisotropy on MPI performance was modeled by Weizenecker *et al.* (*52*) for particles between 20 and 30 nm, where particles with low anisotropies had superior MPI performance compared to those with larger anisotropies. Therefore, anisotropy is also an important parameter to consider when optimizing tracers for MPI. The composition and crystal structure desired should have a low anisotropy, and the synthesis should be controlled to minimize formation of multiple phases and defects that can increase the effective magnetic anisotropy. Both relaxation time and the probability of forming multiple phases and defects increase as nanoparticle size increases. The combination of these challenges results in deviations from the behavior predicted by the Langevin model and hinders improvement of MPI performance with increasing particle diameter. Velazquez-Albino *et al.* (*41*) illustrated this in a high-throughput synthesis approach involving statistical analyses of the effects of particle properties on MPI performance, where deviations from the predictions of the Langevin model and worsening MPI performance correlated to shape anisotropy increasing relaxation effects as tracer physical size increased. These deviations from the ideal model have highlighted its limited ability to describe magnetic nanoparticle behavior in the MPI. More accurate understanding of magnetic nanoparticle magnetization can be obtained from the study of nonequilibrium and nonlinear models, such as the Fokker-Planck equations, to account for the complex dynamics of nanoparticles in MPI (*53*).

## SYNTHESIS AND CHARACTERIZATION OF TRACERS

### Methods of magnetic nanoparticle synthesis

Many methodologies for synthesizing magnetic nanoparticles have been developed over the past several decades. Each synthesis route offers variations in terms of complexity, yield, particle size, size distribution, magnetic properties, morphology, and composition. In the context of particles synthesized for MPI, the most common synthesis methods include thermal decomposition, coprecipitation, and solvothermal methods. Outside of synthetic methods, some researchers have used modified bacterial magnetosomes as MPI tracers (*54*–*57*). Many other techniques for synthesizing magnetic nanoparticles have been used, which may have attractive features, but have not been widely used for the synthesis of MPI tracers (*58*, *59*). Although most of the work to date in MPI has focused on SPIONs, nanoparticles of materials other than iron oxide, such as substituted ferrites, zero-valent iron particles, FeCo, FePt, and iron carbide, may be suitable for MPI, but few studies to date have considered them. The sections below provide further information on the corresponding studies reported to date.

Thermal decomposition methods use relatively high temperatures to decompose organometallic precursors in the presence of organic surfactants and produce magnetic nanoparticles. This method offers the advantages of having a narrow size distribution and high crystallinity. In addition, the method is highly tunable with many parameters that can be changed to control particle size, distribution, shape, morphology, and magnetic properties. Specifically, the decomposition temperature, reaction time, concentration of surfactants, and type of solvents and surfactants are all modifiable and directly affect the properties of the produced nanoparticles (*43*, *60*, *61*). Several recent studies have focused on improving the uniformity of nanoparticles while improving magnetic properties of large (>20 nm) particles (*42*, *62*, *63*). The main disadvantages of the thermal decomposition method are its complexity and sensitivity to reaction conditions, which limits reproducibility, and its reliance on toxic organic solvents and resulting hydrophobic nanoparticles that must be transferred into aqueous media to be used in biomedical applications.

Coprecipitation is a very common synthesis route for magnetic nanoparticles because of its simplicity, scalability, and use of nontoxic and inexpensive materials. In general, metal salts of differing charge, such as Fe^2+^ and Fe^3+^ salts, are dissolved in an aqueous solution and then precipitated by addition of a strong base (*59*). Several reaction parameters, such as pH, reaction temperature, ionic strength, and ratio of ions, can be varied to influence the shape, size, and composition of the particles (*64*). However, the method lacks precise control over nanoparticle size and shape, limiting its usefulness for the synthesis of high-quality nanoparticles requiring uniform size and magnetic properties (*59*, *65*).

The solvothermal or hydrothermal method is common in producing many types of nanocrystals using high pressures and temperatures (*65*). While it has been less explored in producing magnetic nanoparticles for MPI than the other methods, the method is notable for producing materials of high crystallinity (*59*). Magnetic nanoparticles of varying sizes (10 to 40 nm) with narrow size distributions and saturation magnetization values comparable to those of thermal decomposition samples have been reported with this method (*59*, *66*). Particle properties can be controlled on the basis of reaction time, pressure, and temperature. However, this method is generally limited by complexity, because of the high temperatures and pressures required, and the slow reaction kinetics (*59*, *66*, *67*).

### Postsynthesis and in situ synthesis modifications

It is common for synthesized particles to have saturation magnetization values less than that of the bulk material. This is often attributed to the existence of a layer of nonmagnetic material at the surface of the nanoparticles or to the presence of multiple phases or magnetic domains in a single particle (*44*, *60*, *68*–*78*). For SPIONs, one technique for improving magnetic properties is postsynthesis oxidation and annealing, whereby synthesized particles are heated to an elevated temperature for several hours while exposed to atmosphere or optionally bubbling oxygen directly into solution. This method aims to convert nonmagnetic iron oxide phases such as wüstite (FeO) to the preferred magnetite (Fe_3_O_4_) phase, although it could also yield maghemite (γ-Fe_2_O_3_). This has been shown to increase the saturation magnetization and presence of magnetite in nanoparticles. However, as particles increase in size, it requires increasing processing time and may have limited success (*43*, *60*, *79*, *80*). Oxygen may also be provided by the thermolysis of chemicals such as trimethylamine *N*-oxide as an alternative to direct oxidation by oxygen gas (*60*).

Unni *et al.* reported magnetic nanoparticles with uniform magnetization by introducing molecular oxygen during the thermal decomposition synthesis of iron oxide nanoparticles. The resulting defect-free phase-pure magnetite nanoparticles had similar physical and magnetic diameters (*42*). MPI performance was evaluated using a custom-built magnetic spectrometer/relaxometer, revealing improved performance for nanoparticles of similar physical size synthesized with molecular oxygen compared to those synthesized under anoxic conditions. Other studies have used high-resolution TEM to show that nanoparticles synthesized by thermal decomposition under anoxic conditions contain defects and multiple crystal structures within a single particle (*81*–*83*). Chen *et al.* (*61*) also illustrated similar ability to synthesize phase-pure magnetite nanoparticles by incorporating benzyl ether that undergoes radical decomposition to affect the redox potential of the solution. These findings illustrate the importance of rigorous magnetic characterization when optimizing tracers for MPI, as these properties heavily influence the tracers’ performance.

Because the size and size distribution of magnetic nanoparticles are important in determining the performance of tracers in MPI, several techniques have been developed to isolate particles of desired size and to reduce polydispersity after synthesis to improve tracer performance. A common method is using centrifugation to separate particles on the basis of size. This may occur as a single centrifugation step to eliminate aggregates of particles while preserving individual particles (*84*) or may involve fractionation through a series of centrifugation steps (*85*). Density gradient separation has also been used to separate particles in a single centrifugation step, where particles are sorted into separate solutions of varying density (*86*). In place of centrifugation, magnetic fields may also be used to separate particles on the basis of magnetic properties that are expected to vary with size by placing samples in the presence of magnets and separating the resultant supernatant and sediment (*87*). Alternatively, the particles may flow orthogonally to a magnetic field, fractionating samples by capturing larger particles while allowing smaller particles to pass (*88*).

In addition, particle coatings and surface modifications are important for determining tracer colloidal stability and performance in specific biomedical applications. Various materials may be used to improve tracer colloidal stability in specific media or for additional functionalization of tracers. While discussion of specific surface modification strategies is outside of the scope of this review, we encourage interested readers to refer to reviews on this topic (*89*–*91*).

### Particle characterization

**Fig. 3.**
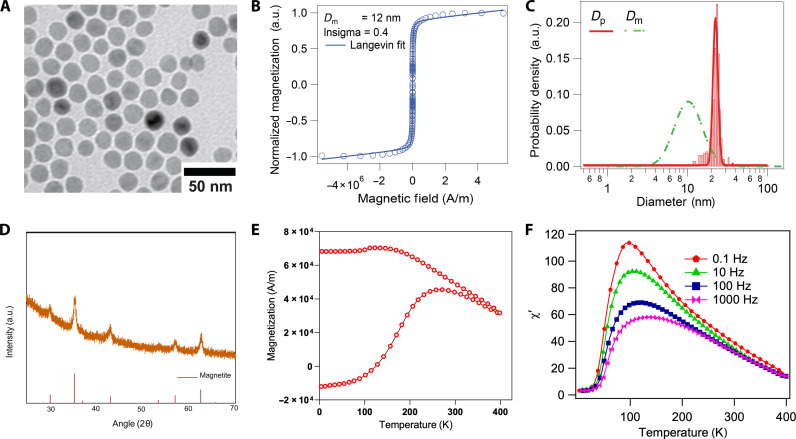
Example data for characterization of SPIONs. (**A**) TEM image of a SPION. (**B**) Magnetization response curve collected by SQUID magnetometry. (**C**) Log-normal physical (*D*_p_) and magnetic (*D*_m_) diameter distributions of a SPION synthesized without oxygen illustrating the discrepancy between the magnetic and physical diameters. (**D**) XRD pattern of a SPION compared against a magnetite reference. (**E**) ZFC/FC magnetization curve of a SPION. (**F**) Temperature-dependent DMS of a SPION at several field frequencies. Reprinted or adapted with permission from (*42*). Copyright 2017 American Chemical Society.

Several physical and magnetic properties are commonly reported in the MPI tracer literature. Physical size is almost always reported and may be determined by several characterization methods. Most commonly, TEM is used to determine particle core size and size distribution as shown in Fig. 3A. TEM may also be used for evaluating crystal structure using selected-area electron diffraction (*43*) and elemental composition using energy-dispersive x-ray spectroscopy (*92*). It is worth noting that TEM characterization involves inherent bias in that the operator determines which regions are captured and aggregates typically cannot be easily analyzed compared to individual particles. The method also collects a very limited number of samples, typically using only hundreds or thousands of imaged particles. This may not be enough to capture the true size and morphology distribution, as 1 mg of 20-nm diameter iron oxide nanoparticles contains approximately 50 trillion individual nanoparticles. Hence, other techniques should be used when available to evaluate polydispersity more fully. Small-angle x-ray scattering (SAXS) is a less frequently reported technique that gives information about nanometer-scale distances including size, shape, and particle spacing (*63*, *93*). Compared to TEM, SAXS samples data from a far greater number of particles, but analysis requires models based on expected particle shape characteristics (*63*, *94*). Dynamic light scattering may be used to assess the hydrodynamic size of particles and evaluate their polydispersity and presence of aggregates, but it lacks the resolution and reproducibility necessary to correlate size to MPI performance, especially in highly magnetic nanoparticles that tend to aggregate (*43*). X-ray diffraction (XRD) is typically used to evaluate the material phase but can also be used to estimate and compare crystallinity of particles on the basis of the peak width, as illustrated in Fig. 3D (*60*), and average crystallite size can be calculated using Scherrer’s equation (*95*). However, XRD is not able to distinguish between phases with a shared chemical structure, such as magnetite and maghemite that share an inverse spinel structure, and has limited ability to identify mixed phases present in small amounts (*42*). Hence, other techniques, such as Mossbauer spectroscopy, are necessary for more complete information on phase composition (*79*, *95*).

Many magnetic properties, including saturation magnetization and susceptibility, are calculated on the basis of magnetization curves obtained from magnetometers, an example of which is displayed in Fig. 3B. Techniques include vibrating sample magnetometry (VSM) and superconducting quantum interference device (SQUID) magnetometry (*42*, *43*, *96*). Particle size and magnetic properties generally affect the MPI performance of a given particle, as predicted by the Langevin function. This raises the important but uncommonly reported parameter of magnetic diameter, which accounts for defects in particles such as the existence of multiple magnetic domains or crystallographic phases that lower MPI performance. The magnetic diameter corresponds to the diameter of a particle that would produce the same signal as measured according to the Langevin function. A magnetic diameter distribution may be obtained by fitting a lognormal weighted Langevin function to a measured magnetization curve (Fig. 3B) as suggested by Chantrell *et al.* (*78*). An example comparing physical and magnetic diameter distributions is shown in Fig. 3C (*42*), demonstrating that they do not necessarily align. Magnetocrystalline anisotropy is another important and often neglected property of magnetic nanoparticles that is relevant in determining the Néel relaxation time and that ultimately affects MPI performance. Anisotropy constants of immobilized samples may be calculated using zero field–cooled/field-cooled (ZFC/FC) magnetization curves or through temperature-dependent dynamic magnetic susceptibility (DMS) measurements, examples of which are given in Fig. 3 (E and F), respectively. ZFC/FC curves are used to determine the blocking temperature while DMS measures relaxation time, both of which can be used to estimate the anisotropy constant by applying the Néel or Vogel-Fulcher model (*97*, *98*). Relatively few studies have reported anisotropy values of magnetic nanoparticles, especially in MPI. However, effective anisotropy values have been reported to be up to an order of magnitude greater than the bulk value for the material and can vary widely between nanoparticle samples (*99*, *100*). It is important to note that sample preparation techniques may also affect measured magnetic properties because of interactions between particles and between particles and the surrounding matrix (*97*, *98*).

**Fig. 4.**
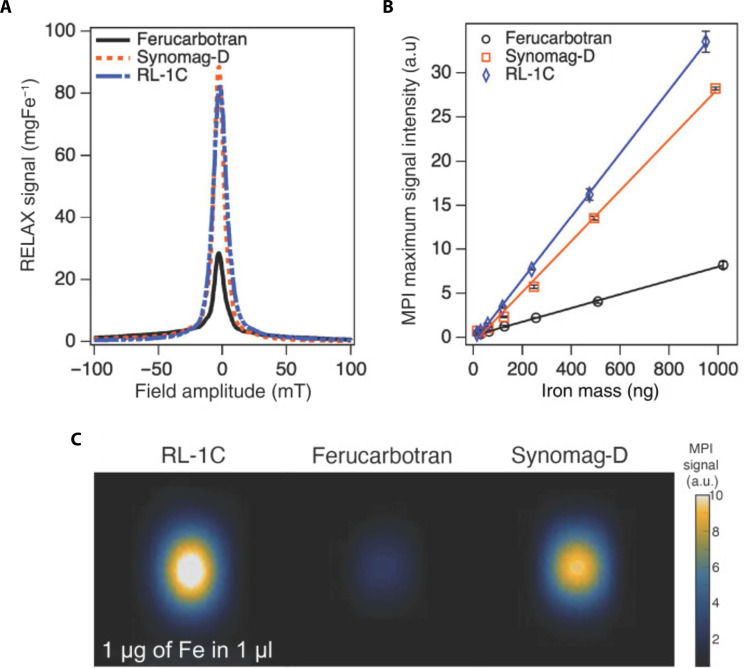
Characterization of MPI properties. (**A**) PSF obtained from RELAX module shows signal intensity normalized by mass of iron, (**B**) serial dilution shows linear relationship between MPI signal intensity and iron mass, and (**C**) MPI 2D maximum intensity projections of the same mass of iron of developed tracer compared to commercially available tracers (*18*).

Tracer MPI performance can be evaluated using an MPI scanner directly, such as the Magnetic Insight MOMENTUM scanner, or using magnetic particle spectrometer (MPS) or magnetic particle relaxometer (MPR) measurements. A MPS uses only an AMF to probe nonlinear regions of particle magnetization and measure tracer excitation frequency and harmonics (*33*, *101*). A MPR builds upon the MPS approach by applying a bias field that changes over time and that simulates movement of the FFR in an MPI scanner, allowing for characterization of relaxation effects that are not measured by MPS (*35*, *101*). These two characterization techniques do not precisely mimic the conditions during an MPI scan because they lack a magnetic gradient field used to encode spatial information (*101*). The main parameters to be measured in MPS, MPR, or MPI are sensitivity and resolution. In the case of MPS, the harmonic spectrum of the particle is measured and can be indirectly related to the signal and resolution of the tracer (*11*, *33*). For x-space MPI, signal intensity is typically reported as a relative value normalized by either the mass of iron or the mass of magnetite of a sample, while resolution is typically reported as the FWHM of the measured intensity versus applied field curve, which is called the PSF. PSFs for several particles are given in Fig. 4A, comparing a research tracer to commercially available magnetic nanoparticles as references. Figure 4B illustrates another method of reporting intensity, in which the signal of a serial dilution of a nanoparticle solution is shown, the slope of which corresponds to the specific signal intensity of the particles. This method shows the expected linear relationship of MPI signal with tracer mass and can also be used to determine the lower limit of detection for a specific tracer. In addition, MPI performance can be characterized by acquiring 2D scans to compare the maximum intensity of different tracers in samples with the same iron mass (Fig. 4C). It should be noted, however, that these properties are functions of the precise magnetic field conditions used in the measurement, such as field gradient (in an MPI scanner) or bias field (in an MPR) and AMF amplitude and frequency. For example, it has been demonstrated that resolution and sensitivity are oppositely correlated with AMF amplitude, meaning that there is a trade-off between resolution and sensitivity when choosing excitation field strength (*102*). It is therefore important to specify the parameters used while measuring MPI performance. In addition, MPS and MPR do not precisely mimic the conditions the tracers experience or capture the acquisition and reconstruction processes of MPI. Hence, the data cannot be expected to be directly translatable between the methods. Because of variation between measurement systems, commonly available commercial nanoparticles are often used as a benchmark for comparison of the relative performance of tracers.

### Commercial tracers

A variety of magnetic nanoparticles are commercially available and have been used as MPI tracers by various research groups. Most of these commercial particles were first synthesized for other applications, such as treating iron deficiency or for use as MRI contrast agents, and later adapted for use in MPI. These particles therefore tend to have suboptimal MPI performance, as noted by Gleich and Weizenecker for Resovist (*11*). Despite their suboptimal performance, they have frequently been used as benchmarks in comparing MPI performance, especially sensitivity, of custom tracers. Resovist is a carboxydextran-coated magnetite nanoparticle that was approved for clinical use as an MRI contrast agent (*103*). Despite the small 3- to 5-nm diameter of its SPIONs, it shows relatively good performance in terms of sensitivity, which is often attributed to particle interactions that generate a small population with an effective magnetic diameter of 25 to 30 nm (*11*, *104*). Ferucarbotran is an off-label version of Resovist with the same formulation (*18*, *105*). VivoTrax is a rebranded version of ferucarbotran sold specifically for use with MPI (*106*), whereas VivoTrax+ is a recently developed version of VivoTrax that is magnetically fractionated to select for the population of more magnetic particles to enhance MPI performance in terms of specific signal and resolution (*106*, *107*). Feraheme (ferumoxytol) is another iron oxide nanoparticle with a carboxymethyl-dextran coating that is Food and Drug Administration approved as a treatment for iron deficiency and is used off-label as an MRI contrast agent (*108*). Direct comparisons have shown Feraheme to have lower sensitivity and poorer resolution compared to VivoTrax (*108*). Other examples include Perimag and Synomag-D, clustered magnetite nanoparticles with a dextran shell of 130 nm and 30- to 50-nm hydrodynamic diameter, respectively, that show higher sensitivity and improved resolution compared to Resovist and VivoTrax (*109*, *110*). PrecisionMRX are 24-nm monodisperse particles in various coatings that differ from other commercial particles that tend to be large polydisperse clusters of small particles, though MPI performance is still comparable (*109*). Table 1 compiles a list of commercial tracers with measurements of physical size, FWHM, and MPI sensitivity. It is noted that many of these results were obtained from MPR measurements, which do not accurately represent actual performance in MPI scans (*111*). The large differences between commercial and custom nanoparticles, discussed further below, reveal that there is much room for optimization.

**Table 1.** Summary of various commercial MPI tracers. Physical and magnetic diameters, MPI performance, measurement system, and conditions. NR, not reported.

Tracer	Physical diameter (nm)	Magnetic diameter (nm)	FWHM	MPI sensitivity	Measurement system	AMF frequency, amplitude, and selection field gradient	Reference
Resovist	4	NR	9.6 mT	13.74 (mV/mg_Fe_)	MPR	20 kHz, 20 mT	(*109*, *132*)
VivoTrax	4.2	NR	11.4 mT	8.83 (mV/mg_Fe_)	MPR	20 kHz, 20 mT	(*109*)
VivoTrax+	NR	NR	7.9 mT	2.4x VivoTrax	MOMENTUM	NR	(*107*)
Ferucarbotran	9.6	7.6, 22.1*	11.2 mT	25.8 (a.u./mg_Fe_)	MOMENTUM	45 kHz, 16 mT, 5.7 T/m	(*18*)
Synomag-D	28.6	8.2, 19.3*	9.2 mT	87.8 (a.u./mg_Fe_)	MOMENTUM	45 kHz, 16 mT, 5.7 T/m	(*18*, *110*)
Feraheme	6–7 / NR	NR	39.5 mT	2.12 (mV/mg_Fe_)	MPR	20 kHz, 20 mT	(*109*, *133*)
PrecisionMRX	24.4 / NR	NR	12.4 mT	13.89 (mV/mg_Fe_)	MPR	20 kHz, 20 mT	(*109*)
Perimag	Clustered / NR	NR	7.3 mT	29.49 (mV/mg_Fe_)	MPR	20 kHz, 20 mT	(*109*)

*Magnetic diameters were determined by fitting a bimodal lognormal size distribution resulting in mean diameters representing two populations.

## RECENT TRACERS ENGINEERED FOR USE IN MPI

### Iron oxide nanoparticles

**Fig. 5.**
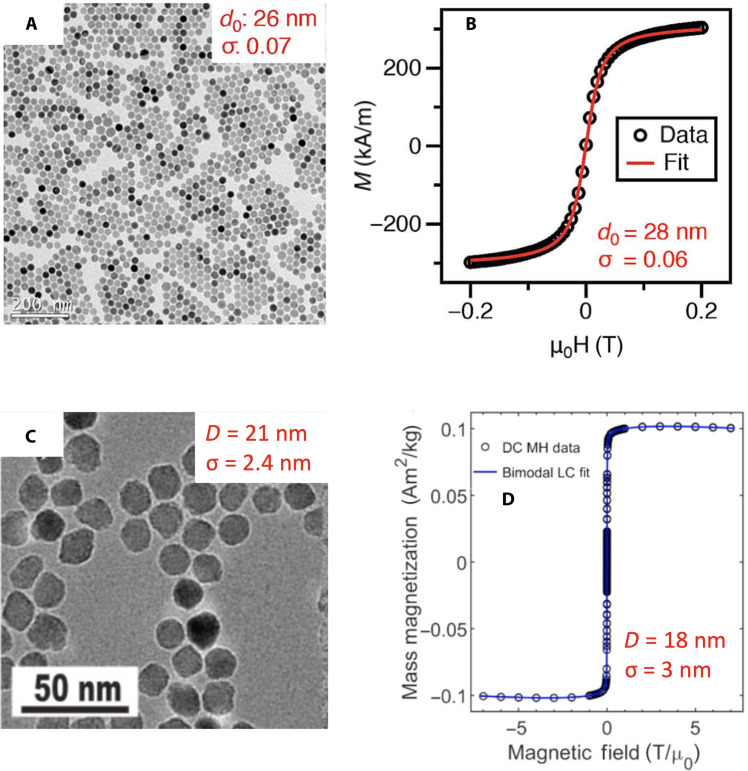
Physical and magnetic size characterization of LS-1 and RL-1 tracers obtained from thermal decomposition synthesis of iron oleate with modifications of postsynthesis and in situ oxidation, respectively, to improve magnetic properties and MPI performance. Evaluation of (**A** and **C**) physical size via TEM and (**B** and **D**) magnetic size from fitting the *M*(*H*) data to the Langevin function for LS-1 and RL-1 tracers, respectively, suggests uniform magnetization. Statistics correspond to a lognormal distribution for LS-1 [(A) and (B)], where *d*_0_ corresponds to the median core diameter and σ correponds to the geometric deviation. Normal distribution statistics are shown for RL-1 [(C) and (D)], where *D* correponds to the mean diameter and σ corresponds to the SD. In comparing the magnetization curves, note the very different reported field amplitude ranges. (A and B) Reprinted with permission from (*111*) Copyright 2014 IEEE and (C and D) adapted from (*18*).

The first tracers used when MPI was developed and reported in 2005 were iron oxide nanoparticles (*11*); therefore, it comes as no surprise that many current efforts focus on improving their performance. To facilitate comparison of recently developed tracers, Table 2 compiles a list including their reported physical and magnetic properties, MPI performance, and measurement parameters. As explained previously, magnetic properties of tracers are key to improving MPI performance, which requires optimization and tailoring of synthesis methods for MPI. Current synthesis modifications to improve magnetic properties of interest were discussed in an earlier section. Both postsynthesis annealing (*43*) and in situ oxidation (*42*) in the thermal decomposition synthesis have demonstrated an improvement in the magnetic properties of nanoparticles by increasing the saturation magnetization closer to that of bulk magnetite and obtaining similar physical and magnetic diameter distributions. The postsynthesis annealing method has yielded 26-nm spherical single-core particles (called LS-1 by the authors; Fig. 5, A and B) with three times better sensitivity than Resovist and improved resolution (*111*), while the in situ oxidation method first reported by Unni *et al.* (*42*) was modified by Liu *et al.* (*18*) to obtain 22-nm spherical single-core iron oxide nanoparticles (called RL-1 by the authors; Fig. 5, C and D) with three times greater sensitivity than ferucarbotran and comparable resolution. Furthermore, both LS-1 and RL-1 tracers were tailored for blood pool imaging with different polyethylene glycol coatings for long-term colloidal stability (*18*, *20*). Hence, both postsynthesis and in situ oxidation modifications to the thermal decomposition synthesis show great promise for improving tracer MPI performance.

**Table 2.** Summary of recently developed MPI tracers. Physical and magnetic diameters, MPI performance, measurement system, and conditions.

Tracer	Physical diameter (nm)	Magnetic diameter (nm)	FWHM (mT/mm)	MPI signal	Measurement system	AMF frequency, amplitude, and selection field gradient	Reference
LS-XXX	26	28	NR / 1.7 mm	3× Resovist	Berkeley MPI	23.2 kHz, gradients (*x*, *y*, *z*) 7, 3.5, 3.5 T/m	(*20*, *22*, *111*, *134*–*139*)
			NR / 0.7 mm		MPS		
RL-1	22.6	18.1	11.9 mT / NR	82.6 (a.u. / mg_Fe_), 3.2× ferucarbotran	MOMENTUM	45 kHz, 16 mT, 5.7 T/m	(*18*)
CIONs-22	22.4	NR	NR / NR	4.15× VivoTrax	MOMENTUM	45 kHz, 5.7 T/m	(*16*)
MCP 3	32.0	NR	NR / 2 mm	5× Resovist	MPS / Bruker MPI	MPS: 25 kHz, 10 mT; Bruker: 25 kHz, 12mT, (1.25, 1.25, 2.5) T/m	(*116*)
Δ*mamJ* magnetosome	NR	NR	NR / 2.98 mm	221.2 (a.u./μg_Fe_)	MOMENTUM	45 kHz, 20 mT, 5.7 T/m	(*54*)
SFMIO	29.6	12.2	0.945 mT / 0.15 mm	0.6 (mV/mg_Fe_)	Berkeley MPI	6.3 T/m	(*121*)
CS20-A	20	NR	NR / NR	3× VivoTrax	MPS	16.8 kHz, 20 mT	(*80*)
NiFe_2_O_4_@PAA	12.1	NR	7.7 mT / NR	NR	MPS	9.9 kHz, 15 mT	(*96*)
ZnFe_2_O_4_/C@PDA	180	NR	NR / 13.2 mm	4.7× VivoTrax	MOMENTUM	NR	(*127*)
FeCo@C-PEG	10.2	NR	NR / 3.1 mm	6× VivoTrax	MOMENTUM	45 kHz, 6 T/m	(*86*)
Fe(0) core-iron oxide shell	14	NR	NR / 1.8 mm	0.8× VivoTrax	MOMENTUM	45 kHz, 6 T/m	(*130*)
MNP@Au	30.6	10.2	NR / NR	14 (mV/mg_Fe_)	MOMENTUM	45 kHz, 20 mT, 6 T/m	(*117*)
MnFe2O4	17.4	NR	11.73 mT / NR	NR	MPR	9.9 kHz, 15 mT	(*95*)
MnFe2O4	8	NR	NR / NR	0.7× VivoTrax	MOMENTUM	45 kHz, 16 mT, 6 T/m	(*92*)
Zn_0.4_Fe_2.6_O_4_	19.1	NR	10.7 mT / NR	5× PrecisionMRX	MPR	16.8 kHz, 20 mT	(*113*)
Zn_0.4_Fe_2.6_O_4_ cubic	15.4	NR	16.4 mT / NR	2× PrecisionMRX	MPR	16.8 kHz, 20 mT	(*113*)
ZnFe_2_O_4_@PAA	18	NR	4.89 mT / NR	NR	MPR	9.9 kHz, 15 mT	(*128*)
ZnMNP-ACM	10	NR	NR / 1.9 mm	NR	Bruker MPI	25 kHz, 12 mT, gradients (*x*, *y*, *z*) 1.25, 1.25, 2.5 T/m	(*118*)
Zn_0.2_Fe_2.8_O_4_ cubic	12	NR	NR / NR	3× VivoTrax	MPR	16.8 kHz, 16 mT	(*114*)
Zn_0.1_Co_0.5_Fe_2.4_O_4_ cubic	11	NR	NR / NR	0.5× VivoTrax	MPR	16.8 kHz, 16 mT	(*114*)
Zn_0.33_Fe_2.52_O_4_	16	12	NR / NR	0.4× Resovist	Bruker MPI	24.5 kHz, 16 mT	(*129*)

**Fig. 6.**
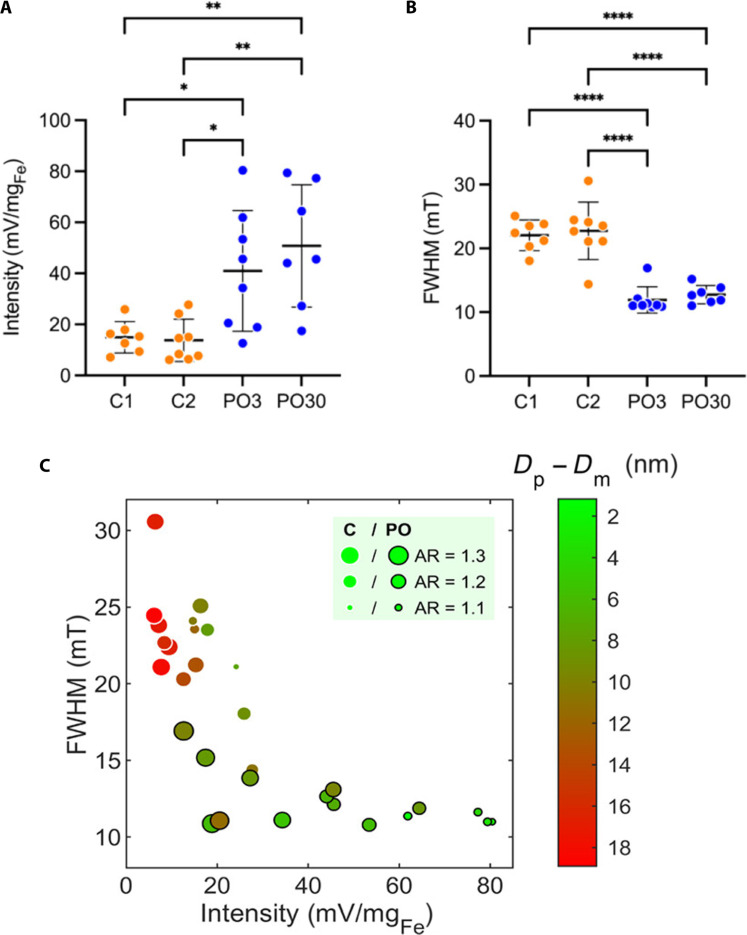
Effect of postsynthesis oxidation of SPIONs synthesized in anoxic conditions in an experimental setup that allowed for eight reaction replicates per synthesis condition. Two control groups (C) and two postsynthesis oxidation groups (PO) were compared. The work highlights improvement alongside variability in MPI performance: (**A**) signal intensity (from top to bottom **P = 0.0041, **P = 0.0022, *P = 0.0394, and *P = 0.0233) and (**B**) FWHM. ****P < 0.0001. (**C**) Enhancing MPI performance requires consideration of nanoparticle properties such as the discrepancy between physical and magnetic diameter (*D*_p_ − *D*_m_) and the physical shape (i.e., AR). Reproduced with permission from (*41*). Copyright 2024 American Chemical Society.

Despite tremendous progress in chemical synthesis of nanomaterials to control their size, shape, and functional properties, issues of poor reproducibility plague the field (*112*). This motivated Velazquez-Albino *et al.* (*41*) to study the effect of postsynthesis oxidation on MPI performance of iron oxide nanoparticles synthesized via thermal decomposition of an iron oleate precursor. Results showed that the postsynthesis oxidation treatment leads to improvement in magnetic properties, such as saturation magnetization and magnetic diameter, enhancing MPI performance. However, the study also highlighted the variability in nanoparticle properties and MPI performance (Fig. 6, A and B), the limited effectiveness of the postsynthesis oxidation treatment to improve MPI performance, and the challenge of worsening performance due to increasing relaxation effects. The work elucidated that the discrepancy between physical and magnetic diameters (*D*_p_ − *D*_m_) and the nanoparticle’s aspect ratio (AR), a measure of deviation from spherical shape, heavily affect MPI performance (Fig. 6C), indicating the need for more reproducible synthesis methods that allow for fine control of physical and magnetic properties to achieve optimal MPI perfomance.

Although interest in MPI steadily grows, there is a scarcity of MPI scanners, mostly because of their novelty, complexity, and cost. This has resulted in studies that claim tracers tailored for MPI without actually measuring performance in a MPI scanner. Ferguson *et al.* (*111*) probed differences in performance in MPI scanners with system matrix and x-space reconstruction algorithms compared to measurements in an MPS. Similar relative improvement to Resovist was observed for both system matrix and x-space MPI reconstruction, yielding two to three times greater signal intensity; however, they did not report signal per mass values for further comparison. LS-1 resolution was estimated from the FWHM of the PSF phantom image to be 1.6 mm, which agreed with the ability to resolve phantoms separated by 1.7 mm. On the other hand, the estimated resolution from MPS measurements was 0.7 mm, an overestimate that is not yet understood. Table 2 summarizes recently developed tracers to facilitate comparison while highlighting the different measurement systems and conditions used. The tracer with the best MPI performance reported so far is believed to be LS-1 (*111*), synthesized by the small startup Lodespin Labs, that closed business in 2017. Iterations of this tracer have been used in numerous MPI studies under various names, such as LS-13, LS-017, and LS-008. Therefore, we have labeled it as LS-XXX in Table 2.

Cubic-shaped iron oxide nanoparticles synthesized by thermal decomposition have also been studied for use as MPI tracers for various applications (*16*, *80*, *105*, *113*, *114*). Wang *et al.* (*16*) compared different-sized cubic nanoparticles to spherical nanoparticles and reported that 22-nm edge length cubic nanoparticles (CIONs-22) had superior MPI performance in a MOMENTUM scanner, with a 4.15-fold higher sensitivity than Vivotrax. They attribute worse MPI performance of other nanoparticles in the study to increased relaxation for the larger-sized cubic nanoparticles, and the presence of wüstite in 22-nm spherical particles through XRD. On the other hand, another study compared spherical and cubic particles of different sizes with and without a zinc dopant and found that spherical particles had superior MPI performance than cubic particles measured on an x-space MPR (*113*). In this case, nanoparticles had a similar crystal structure, as particles of both were determined to be magnetite through XRD. The disagreement between these studies highlights how differences in MPI performance are difficult to attribute solely to one nanoparticle property—such as shape in this case, especially when using a limited number of samples. Studying the effect of nanoparticle shape or composition in MPI can be challenging, as the effect is often confounded with differences in particle size, crystallinity, anisotropy, relaxation mechanism, and iron oxide phases. Another study of magnetic nanostructures composed of iron oxide nanocubes also highlights the effects of interactions, as small clusters containing two or three nanocubes showed superior MPI performance than single nanoparticles, large clusters, and Vivotrax (*105*). More recently, Sojková *et al.* (*80*) reported 20-nm nanocubes composed of mostly Fe_3_O_4_ (CS20-A) synthesized by thermal decomposition, starting from FeO/Fe_3_O_4_ core-shell nanocubes and subjected to a postsynthesis thermal treatment, yielding tracers with three times the signal of VivoTrax measured with a custom MPS. Extending work beyond nanocubes, Nigam *et al.* (*115*) synthesized iron oxide nanorods and compared them to spherical iron oxide particles to explore the impact of shape anisotropy on MPI performance. Although they show improved sensitivity of the rod-shaped particles, the particles have notably different sizes, with spherical particles of 12-nm diameter compared to rods of 8-nm diameter and 50-nm length. In addition, little magnetic characterization was performed beyond showing a slight increase in saturation magnetization. Hence, it is difficult to isolate the effects of shape anisotropy from other particle properties in the study.

Multicore nanoparticles are also of interest for MPI applications. The commercial multicore tracer Synomag has been reported to have three times better sensitivity than Resovist in a traveling wave MPI (*110*). Multicore particle (MCP 3) tracers have been prepared by Kratz *et al.* (*21*, *87*, *116*) via coprecipitation synthesis, performing approximately five times better than Resovist when measured with magnetic particle spectroscopy and phantom MPI scans in the Bruker MPI scanner. Recently, core-shell nanoparticles have also been explored for MPI, where structures such as magnetite-gold core-shell nanoparticles are applied as multimodal imaging probes (*117*). The addition of the gold shell is reported to slightly lower the saturation magnetization but slightly increase the magnetic diameter and MPI intensity relative to uncoated iron oxide particles and is claimed by the authors to be due to interactions between the magnetite core and gold shell surfaces.

Other efforts focus on engineering iron oxide nanoparticles into composites for different applications. For example, Janus nanoparticles were prepared by encapsulation of iron oxide nanoparticles with three times the sensitivity of the commercial tracer Vivotrax in semiconducting polymers for cell tracking (*15*). Genetically modified magnetotactic bacteria have also been investigated as living multimodal contrast agents for both MPI and bioluminescent imaging (*54*). For drug delivery and quantification via MPI, a composite consisting of magnetite cores with a doxorubicin loaded PLGA coating was formulated (*26*). Other composites studied for MPI applications include Synomag-D encapsulated into red blood cells for prolonged circulation time (*19*) and artificial chylomicrons for quantification of lipid uptake (*118*). These studies show promise of future synergistic effects of MPI on other medical applications of magnetic nanoparticles and brings interest to further enhancing these effects with multimodal imaging tracers (*119*, *120*).

In an exciting direction for the field, Tay *et al.* (*121*) reported an order-of-magnitude improvement in MPI sensitivity and resolution with superferromagnetic nanoparticle chains measured in a custom 6.3-T/m MPI. The physics behind signal generation of this tracer are different than the MPI theory explained earlier in the review, as chain formation behavior gives rise to a strong dipole reversal when the dynamic coercive threshold of dipole-dipole fields from adjacent nanoparticles in the chain is overcome by the applied field (*122*). This new tracer results in a sharp signal peak, showing great promise for MPI. Further studies are needed to fully understand and characterize these interactions, such as quantitative measurements of remanence decay as reported by Fung *et al.* (*123*). These studies spark interest in understanding systems that deviate from ideal Langevin behavior (i.e., interacting systems) to maximize their potential for enhancing MPI performance. Furthermore, to date, this phenomenon has only been reported for tracers dispersed in toxic organic solvents, suggesting the need for novel formulations that are suitable for biomedical imaging.

### Substituted ferrites and other compositions

MPI tracers of compositions other than iron oxide have also been studied in recent years, though their biocompatibility remains less understood (*40*, *124*, *125*). Most common are substituted ferrites that have a composition of MFe_2_O_4_, where M is a transition metal such as manganese, zinc, nickel, or cobalt. These particles have the same crystal structure as magnetite but have modified magnetic properties that may be beneficial for their performance in MPI. For example, bulk manganese ferrite has a lower magnetocrystalline anisotropy constant than magnetite (3 kJ/m^3^ versus 11 kJ/m^3^), which would be expected to reduce relaxation effects in tracers (*126*). As discussed previously, however, magnetic properties including anisotropy can vary widely between samples and often differ from bulk values, which means that experimental realizations do not always reflect the expected improvements in MPI performance. In the case of manganese ferrite particles, Dogan *et al.* (*95*) synthesized and compared magnetite and manganese ferrite particle properties and MPI performance. Although the manganese particles demonstrated higher saturation magnetization and lower relaxation times, the FWHM were larger than those of the ferrite and commercial particles Perimag and Vivotrax. However, the study lacked size distribution characterization and MPI signal intensity information. Du *et al.* (*92*) similarly compared synthesized magnetite and manganese substituted ferrite particles of 8- and 18-nm diameter and similar size distribution. Manganese substituted ferrite particles demonstrated increased saturation magnetization and decreased magnetocrystalline anisotropy but substantially lower MPI signal intensity compared to the magnetite counterparts. However, measurements of resolution were not reported. Neither group offers explanations for why the manganese ferrite particles show performance that is worse than expected. Although manganese ferrite is a promising theoretical material for MPI tracers, these studies suggest that more work is needed to achieve this theoretical performance in practice.

Zinc ferrite particles contrast with manganese ferrite in recent success in MPI performance. Jiang *et al.* (*127*) compared several very large substituted ferrite nanoparticles synthesized by the pyrolysis of an iron metal organic framework optionally containing zinc, manganese, or cobalt and coated with polydopamine (termed MFe_2_O_4_@C/PDA). The zinc ferrite particles had the lowest measured saturation magnetization but had an MPI signal about 2 times that of all the other samples and 4.7 times that of the commercial particle Vivotrax. However, the particles had a measured FWHM of 13.2 mm compared to 12.4 mm for Vivotrax with unreported AMF and gradient field parameters. The authors give little detail in measurement descriptions and no explanation of the extreme variation in measured FWHM of Vivotrax in comparison to most other studies, which makes comparative evaluation of these particles difficult. Another study reported zinc ferrite particles synthesized in a hydrothermal and coprecipitation process coated in polyacrylic acid with an FWHM of 4.89 mT (1.13 mm) compared to an FWHM of 9.05 mT (2.1 mm) for Vivotrax as measured by MPR, the lowest value reported for a single-core superparamagnetic nanoparticle MPI tracer so far (*128*). However, the custom MPR used has not been validated against MPI system measurements, so it is not clear how well this tracer would perform in an actual MPI scanner. Silvestri *et al.* (*114*) evaluated the performance of nanocubes of varying compositions of zinc- and cobalt-substituted ferrite. Results indicate that the zinc ferrite nanocubes have the highest sensitivity compared to magnetite nanocubes and VivoTrax, while zinc cobalt ferrite has much weaker signal and cobalt ferrite has negligible signal. The zinc ferrite was also claimed to have the best resolution although no direct measurement of FWHM was reported. Kaman *et al.* (*129*) compared zinc and cobalt ferrite nanoparticles synthesized via thermal decomposition, hydrothermal, and solvothermal methods. Their results suggest that zinc ferrite has superior MPI performance compared to cobalt ferrite, regardless of the synthesis method and the silica surface coating thickness. However, the signal is only 40% that of Resovist gauged from the maximum intensity projection of the 3D volume data. Irfan *et al.* (*96*) synthesized nickel ferrite particles coated in polyacrylic acid and measured an FWHM between that of commercial particles Vivotrax and Perimag using a custom MPR.

Other particles that differ from both iron oxide and substituted ferrites have been evaluated for potential as MPI tracers. Song *et al.* (*86*) synthesized carbon-coated FeCo nanoparticles of 10-nm diameter that showed 6 and 15 times greater MPI signal compared to commercial particles VivoTrax and Feraheme, respectively, at the same molar concentration. The authors do not measure FWHM directly but state that two samples can be resolved at 3.1 mm. Another study reported that zero-valent iron core-iron oxide shell nanoparticles achieved 80% of Vivotrax’s sensitivity (*130*). The authors claim a similar spatial resolution as Vivotrax in a MOMENTUM scanner but report a calculated resolution of 1.8 mm compared to 1.1 mm for Vivotrax.

## DISCUSSION AND RECOMMENDATIONS FOR FUTURE WORK

**Fig. 7.**
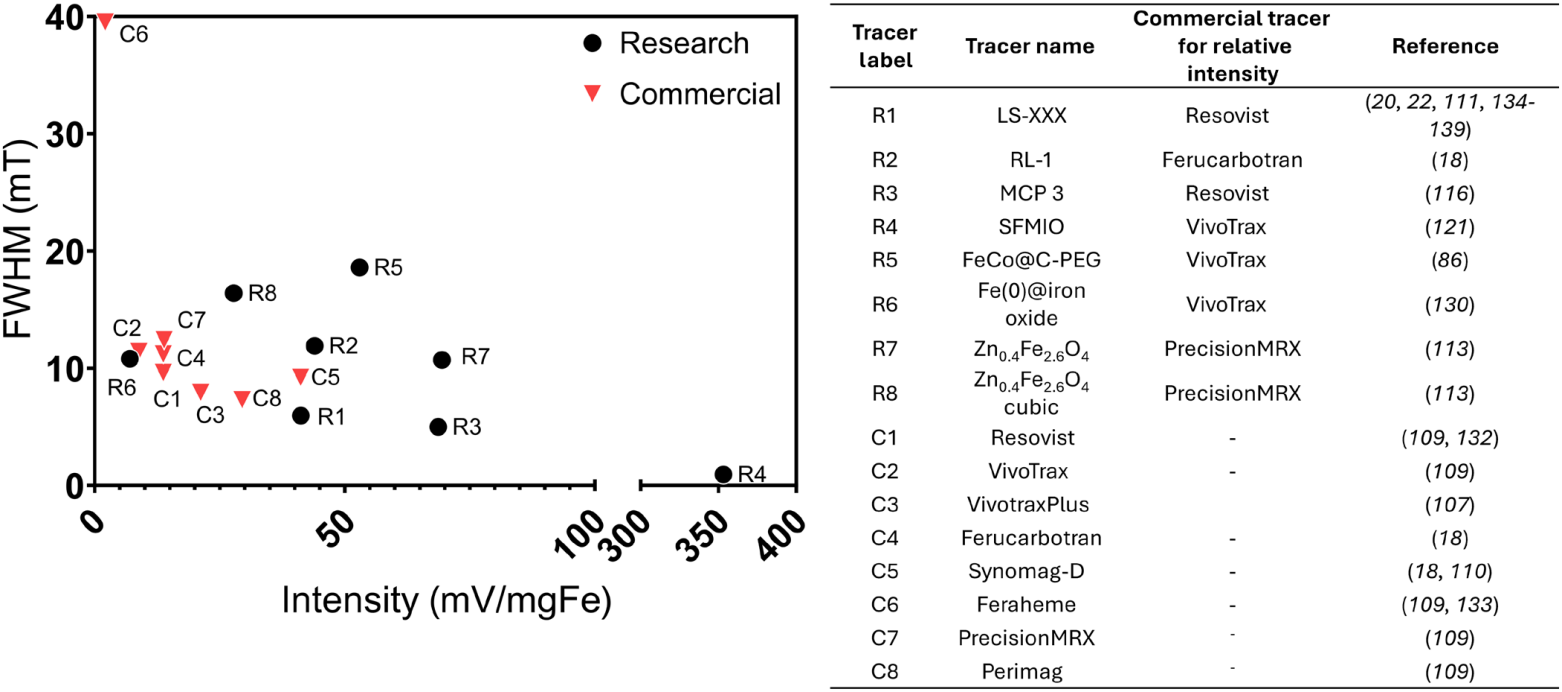
Comparison of MPI signal and resolution (as intensity and FWHM) for research tracers from the literature and for commercial tracers. Specific intensity values of all research tracers were obtained by normalizing to a commercial tracer with a known specific intensity. FWHM reported in spatial units were converted to milliTesla based on the reported magnetic gradient used in the measurement.

The work highlighted here illustrates important advances toward improving tracer performance in MPI. However, meaningful comparison between tracers remains a challenge for the field. Figure 7 compares the intensity and resolution of commercial tracers and selected tracers from literature in a single plot. Many of the reported tracers from Table 2 are not present on the graph because of missing intensity or resolution values. This was due to the value not being reported entirely or only being shown graphically, which hinders comparison of tracers. The specific intensity values used in Fig. 7 are primarily based on the work of Chandrasekharan *et al.* (*109*), where several commercial particles were compared on a single MPS to give specific values of intensity on a unified scale. Relative intensity measurements of an experimental tracer against a commercial tracer could then be converted into specific values on this scale, allowing for visualization of the relative performance of tracers from separate studies. However, this assumes that different imaging instruments and parameters give consistent relations among tracers, for which there is evidence against (*111*, *131*). It should also be considered how the processes of MPR and MPS cannot be expected to directly represent MPI because of differences in the conditions the tracers experience and reconstruction processes required in MPI. Hence, it is critical that these limitations are taken into account and that there is thorough reporting of the conditions used to characterize MPI tracers.

We find that reports of MPI tracers lack consistency in particle characterization and evaluation of tracer performance. Hence, to improve consistency and comparability, we recommend a series of characterization studies that should be performed when reporting a new tracer for MPI. Most important is the performance of a tracer in MPI through its measured sensitivity and resolution. Because of the relative nature of sensitivity to different system parameters, comparison to a reference particle on the same instrument is important (*96*, *113*, *118*, *128*). Some authors neglect to provide direct evaluation of FWHM or sensitivity measurements and use only qualitative descriptions. This inconsistency causes independent comparison of tracers to be difficult. Particle size, including physical and magnetic diameter, is essential to understanding the performance of MPI tracers. Calculation of magnetic diameter distribution is often neglected, which limits understanding of the quality of particles that may contain multiple phases or magnetic domains that can go undetected by more common characterization methods, such as XRD. Hydrodynamic size, relaxation mechanisms, and magnetic anisotropy are also relevant for understanding tracer performance. Recommended characterization criteria to be collected and reported with new MPI tracers is displayed in Table 3. We also encourage the reporting of detailed procedures used in characterization, such as sample concentration and volume used, as well as instrument measurement parameters. In addition, the reporting or availability of tabularized data, especially from magnetic measurements, is suggested to aid in research efforts to predict the magnetic properties of tracers.

**Table 3.** Tracer characterization criteria for new MPI tracers.

Property	Characterization method	Data and meta-data to report	Relevance
Physical size (nm) and morphology	• TEM for size and morphology	• Particle count	Essential
	• SAXS if available	• Size distributions	
Saturation magnetization and magnetic diameter (nm)	• Magnetization curve (VSM or SQUID magnetometry) at room temperature	• Magnetization and applied field as table of values	Essential
	• Fits to appropriate models to obtain magnetic diameter distribution	• Magnetic size distributions	
MPI sensitivity	• Signal in 2D MPI scans for a dilution series to obtain limit of detection	• Excitation field amplitude and frequency	Essential
	• Signal intensity normalized by Fe mass obtained from MPR PSF	• Gradient or bias field amplitude	
		• Particle concentration and volume	
MPI resolution (mT or mm)	• Line scans of 2D images in MPI scanner. Include MPI scan amplitude and gradient	• Excitation field amplitude and frequency	Essential (PSF for x-space MPI and/or line scans)
		• Gradient or bias field amplitude	
	• FWHM of PSF obtained from MPR or MOMENTUM RELAX module	• Particle concentration and volume	
		• Dimensions of vessel	
Comparison to well-characterized tracer	• Examples: Resovist, VivoTrax, and ferucarbotran	• Particle concentration and sample volume	Essential
Hydrodynamic size (nm)	• Dynamic light scattering	• Hydrodynamic size distributions	Strongly recommended
		• Number and length of scans	
Magnetic anisotropy	• ZFC/FC magnetization curves (VSM or SQUID)	• Magnetization and temperature as table of values	Strongly recommended
	• DMS versus temperature measurements	• Particle concentration	
Crystal phase	• XRD	• Diffractogram	Recommended
	• Mossbauer spectroscopy if available	• Angular measurement velocity and resolution	
Relaxation mechanism contributions (Brownian and Néel)	• DMS versus frequency measurements	• Susceptibility, temperature, and frequency as table of values	Recommended
	• MPI measurements in liquid and solid matrix		

In summary, MPI is a rapidly evolving field, driven by advancements in nanoparticle tracer development. This review has showcased progress made in synthesizing iron oxide and other magnetic nanoparticles, resulting in tracers with enhanced MPI performance. The key takeaway is the critical role of magnetic properties in MPI, underscoring the need for comprehensive characterization to push the boundaries of tracer development. While we highlight lack of standardization in tracer characterization, this is not unexpected in a young and rapidly growing field. For example, variability in tracer performance across different measurement systems, such as preclinical MPI scanners and custom instruments, is to be expected. This variability, while challenging, can provide insight as to how tracers behave under different conditions if researchers adopt more detailed characterization and reporting standards. To streamline progress and enable more effective comparisons, we propose standardized characterization criteria as outlined in Table 3. We hope this standardization is a step toward harmonizing research efforts and accelerating advancements in MPI.

## Supplementary Material

20250108-1

## Notes

**Correction (14 January 2025):** Due to a production error, the original version of Fig. 7 cited the incorrect reference numbers. The figure has been updated. The XML and PDF versions of the article have been updated.
